# Impact of Mismatch Angle on Electronic Transport Across Grain Boundaries and Interfaces in 2D Materials

**DOI:** 10.1038/s41598-017-16744-0

**Published:** 2017-11-29

**Authors:** Arnab K. Majee, Cameron J. Foss, Zlatan Aksamija

**Affiliations:** University of Massachusetts–Amherst, Department of Electrical and Computer Engineering, Amherst, 01003-9292 USA

## Abstract

We study the impact of grain boundaries (GB) and misorientation angles between grains on electronic transport in 2-dimensional materials. Here we have developed a numerical model based on the first-principles electronic bandstructure calculations in conjunction with a method which computes electron transmission coefficients from simultaneous conservation of energy and momentum at the interface to essentially evaluate GB/interface resistance in a Landauer formalism. We find that the resistance across graphene GBs vary over a wide range depending on misorientation angles and type of GBs, starting from 53 Ω *μ*m for low-mismatch angles in twin (symmetric) GBs to about 10^20^ Ω *μ*m for 21° mismatch in tilt (asymmetric) GBs. On the other hand, misorientation angles have weak influence on the resistance across MoS_2_ GBs, ranging from about 130 Ω *μ*m for low mismatch angles to about 6000 Ω *μ*m for 21°. The interface resistance across graphene-MoS_2_ heterojunctions also exhibits a strong dependence on misorientation angles with resistance values ranging from about 100 Ω *μ*m for low-mismatch angles in Class-I (symmetric) interfaces to 10^15^ Ω *μ*m for 14° mismatch in Class-II (asymmetric) interfaces. Overall, symmetric homo/heterojunctions exhibit a weak dependence on misorientation angles, while in MoS_2_ both symmetric and asymmetric GBs show a gradual dependence on mismatch angles.

## Introduction

Graphene, a monolayer of sp^2^ hybridized carbon atoms arranged in a honeycomb lattice structure, has a unique Dirac cone electronic structure and exhibits numerous interesting properties including quasi-ballistic electron transport up to several microns of length even at room temperature. Besides graphene, transition metal dichalcogenides (TMDs) are another class of two-dimensional (2D) materials which have attracted intense research interests in recent years. The potential applications of graphene and TMDs have motivated mass scale production of large-area films. Among the most popular methods, chemical-vapor deposition (CVD) on transition metal substrates is relatively cheap and extensively used to grow high quality large two-dimensional sheets^1^. However, CVD-grown films are typically found to be polycrystalline in nature, consisting of many single crystalline grains each with random crystal orientation and separated by grain boundaries (GBs)^2^. Several studies have reported that grain boundaries in 2D materials impact both their electronic^1,3–7^ and thermal properties^8–10^.

The earliest of these studies focused on the electrical resistance across graphene GBs. Experimentally, graphene GB resistance has been found to vary over a broad range from a few Ω *μ*m^11^ to tens of kΩ *μ*m^3,4^. Huang *et al*.^12^ showed a wide distribution of misorientation angles between adjacent grains in a polycrystalline monolayer graphene sheet with a preferential low angle growth of about 7°. The GB resistance across such GBs was found to be about 240 Ω *μ*m. Contrasting this to the sheet resistance of 700 ${\rm{\Omega }}/\square$ for the entire device, they concluded that the GB resistance is about one-third of the total resistance of a 250 nm grain. Koepke *et al*.^13^ observed a reduction in mobility in CVD-grown graphene and attributed it to the strong carrier scattering at grain boundaries. Clark *et al*.^14^ found resistance across graphene GBs to be varying between 40–140 Ω *μ*m for samples with misorientation angles ranging from 9° to 21°. The resistivity of GBs was more than 3 times the bulk resistivity of the grains consistently across all of their samples. There was a positive correlation between misorientation angles and GB resistance, but the width of the transition region surrounding the GB also played a role.

Besides experimental measurements, there are several theoretical studies^15–19^ which have helped to gain more insight on transport across graphene GBs. Yazyev and Louie^15^ found that GBs across grains represented by the same translational vectors are highly transparent to charge carriers with a transmission of about 80%, whereas GBs formed by grains with different translational vectors behave as perfect reflectors of carriers. Vancso *et al*.^16^ performed wave packet dynamical transport calculations to show that transmission properties across graphene GBs depend on misorientation angles as well as localized structures at the boundaries. Zhang *et al*.^17^ showed that intrinsic (defect-free) GBs are almost transparent to carrier transport in highly symmetric GBs. They concluded that the degradation in transmission mainly comes from the extrinsic defects at the boundaries which results in the passivation of the *π*-orbital. Recently, Sun *et al*.^18^ investigated electrical properties along different transport directions with respect to the GB direction using Density Functional Theory (DFT) calculations combined with Green’s function technique. They showed that the zero band gap nature of graphene bandstructure remains intact even in the presence of GBs. They also found that there is an at least 50% current suppression in the transport across GBs as compared to the current in pristine graphene. Despite the numerous studies on various types of graphene GBs, the dependence of GB resistance on misorientation angles is still inconclusive.

There has also been a growing interest in electrical transport of CVD-grown MoS_2_^20–25^; however, little is known about the impact of misorientation angles on its GB resistance. Najmaei *et al*.^20^ studied the individual and collective effect of GBs on electronic transport properties and found that the carrier mobility shows a weak dependence on channel length up to 75 *μ*m. Kang *et al*.^21^ also reported a similar dependence of field-effect mobility on channel length, again indicating that GBs don’t significantly degrade the electronic transport properties in CVD-grown MoS_2_. This observation was further corroborated by Schmidt *et al*.^22^, where they demonstrate that the electronic properties of CVD-grown monolayer MoS_2_ are comparable to those of their exfoliated counterparts. In contrast, Ly *et al*.^25^ showed that MoS_2_ sheets exhibit very poor electrical transport properties (mobilities below 70 cm^2^ V^−1^ s^−1^) for all their devices with different misorientation angles. They observed a positive but non-linear correlation between field-effect mobility and misorientation angles.

Electronic transport in lateral^26–29^ as well as vertical^30–32^ 2D heterostructures has recently gained significant research attention with particular focus on graphene-contacted MoS_2_ lateral (in-plane) heterostructures^33–36^. Graphene has been reported to form an ohmic contact with MoS_2_^26,37^, resulting in an increase in mobility up to an order of magnitude as compared to that of in metal-MoS_2_ field-effect transistors (FETs). This calls for investigating the role of misorientation angles in determining the graphene-MoS_2_ interface resistance in such heterostructures. Throughout the numerous studies of the resistance of GBs and interfaces, a common thread is that the resistance spans a wide range of values depending on mismatch angle. A definitive trend explaining this variation, especially in MoS_2_ GBs and graphene-MoS_2_ interfaces, still requires further investigation.

In this paper, we focus on the fundamentals behind the impact of grain misorientation angles in 2D homojunctions and heterojunctions. Starting from electronic structure obtained through first principles Density Functional Theory (DFT), we calculate the transmission coefficients and boundary/interface resistances for graphene and MoS_2_ grain boundaries, as well as graphene-MoS_2_ heterojunctions. We use the transmission coefficients to compute the conductance of the boundaries/interfaces as a function of both mismatch angle and carrier concentration. In Sec. 2 we further detail our approach and delineate two different classes of GBs (twin and tilt homojunctions) and interfaces (Class-I and II heterojunctions). In Sec. 3, we discuss our results showing that transport across twin homojunctions and Class-I heterojunctions show a weak dependence on mismatch angles, whereas the resistance across tilt homojunctions and Class-II heterojunctions exhibits a strong dependence on mismatch angles. We conclude in Sec. 4 that GBs play a moderate role in MoS_2_ due to its parabolic bands, but can be quite significant in graphene and large-mismatch graphene-MoS_2_ heterostructures owing to graphene’s steep linear Dirac cones.

## Theoretical approach

To study the impact of misorientation angles on interface resistance, we have developed a numerical model based on first-principles DFT electronic bandstructure calculations and electron transmission coefficients from simultaneous energy and momentum conservation. The latter is an extension of the approach originally proposed by Yazyev and Louie^15^ to calculate the transmission coefficient of electrons across a graphene grain boundary. The interface resistance is calculated in the following steps: bandstructure calculations for graphene and MoS_2_ individually from the first principles, rotation of the Brillouin zones (BZ) to account for the misorientation angle between adjacent grains, calculation of electron transmission across the interface from the energy and momentum conservation, and finally computing the interface resistance in the Landauer formalism. For heterojunctions between dissimilar materials, an additional second step involves band alignment at the interface based on the Schottky-Mott rule.

First, we calculate the electronic bandstructure for single-layer graphene and MoS_2_ individually from first principles using Density Functional Theory (DFT) as implemented within the open-source distribution Quantum Espresso^38^ (further details on the DFT calculations are given in the Methods). It is followed by the alignment of the bands at the interface. In homojunctions such as graphene-graphene GBs and MoS_2_-MoS_2_ GBs, the bands are always well-aligned at the interface, whereas in heterojunctions, such as the graphene-MoS_2_ GBs, the bands need to be aligned. In contrast to the planar charge in a 3D interface, a 2D heterojunction forms a line dipole at the junction^34,39^. It has been shown that in 2D heterojunctions, the effect of this interfacial dipole vanishes when the overall dimensions of the device are much larger than the characteristic junction-width, typically about 10 nm^39^. As a result, the band alignment in 2D heterojunctions is far less sensitive to the interfacial details and the band alignment closely follows the Schottky-Mott rule^39^.

In our case, the graphene and MoS_2_ are treated as semi-infinte, so we use the Schottky-Mott rule^40^ and align the vacuum levels of the two materials at the interface. Next, the work function of graphene (*ϕ*_*graphene*_ = 4.55 eV^41^) and the electron affinity of MoS_2_ (${\chi }_{Mo{S}_{2}}=4.2$ eV^42^) are used to align the respective bands away from the interface relative to the vacuum level. Due to the difference in the work function of graphene and electron affinity of MoS_2_, an energy barrier ${{\rm{\Phi }}}_{B}({n}_{C})={\varphi }_{graphene}({n}_{C})-{\chi }_{Mo{S}_{2}}$ is formed at the interface. As graphene is essentially metallic, the bands bend on the MoS_2_ side near the interface to account for the energy barrier height in equilibrium. The amount of band-bending on MoS_2_ side, which is also a function of carrier concentration *n*_*C*_, is given by ${{\rm{\Phi }}}_{interface}({n}_{C})={\varphi }_{Mo{S}_{2}}({n}_{C})-{\chi }_{Mo{S}_{2}}-{{\rm{\Phi }}}_{B}({n}_{C})$ ^26^. The carrier concentration typically depends on the level of impurities, doping, or gating^43–45^.

**Figure 1:**
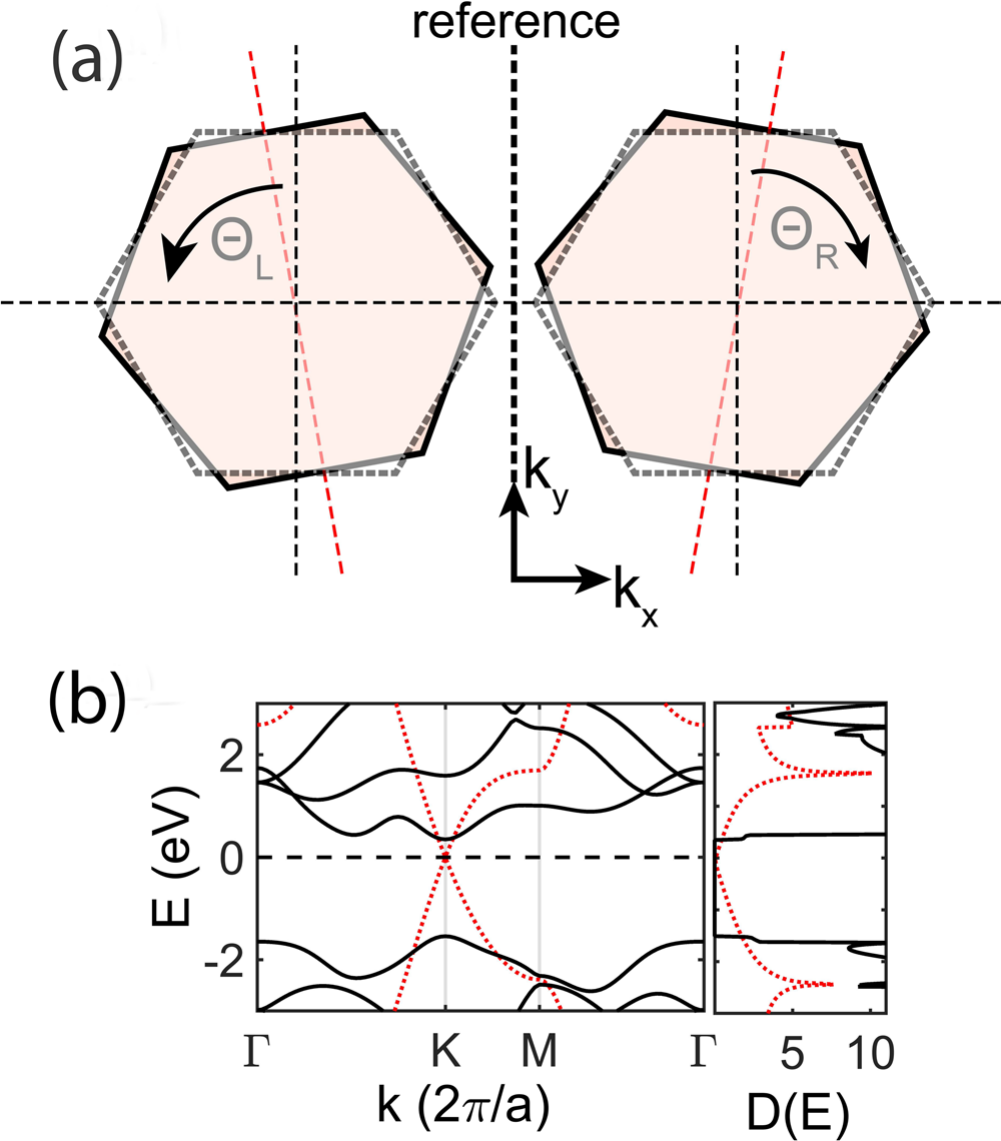
(**a**) Shows orientation of the grains with respect to the interface. The dash-outlined hexagons represent the orientation of the Brillouin zones for perfectly matched condition (Θ_*L*_ = Θ_*R*_ = 0°). Θ_*L*_ is the angle of rotation, measured in anticlockwise direction, between the rotated left grain (solid-outlined hexagon) and the one for perfectly-matched condition (dash-outlined hexagon). Θ_*R*_ is the angle of rotation, measured in clockwise direction, between the rotated right grain and the grain for perfectly-matched condition. The total misorientation angle is then given as Θ_*M*_ = Θ_*L*_ + Θ_*R*_. (**b**) Shows the bandstructure and density of states of graphene (in red) and MoS_2_ (in black) computed from the first principles.

The orientation of the grains with respect to the GB/interface is defined by two angles Θ_*L*_ and Θ_*R*_, each being the angle of rotation between the grain on the left and the right side with respect to the interface, taken here as reference, as shown in Fig. 1. According to our convention, Θ_*L*_ is taken to be positive for anticlockwise rotation of the left grain, whereas Θ_*R*_ is positive for clockwise rotation of the right grain. We define misorientation angle as Θ_*M*_ = Θ_*L*_ + Θ_*R*_. To include the effect of the misorientation angle in our calculation, the wavevectors in the first Brillouin zone are rotated by Θ_*L*_ for the left grain and Θ_*R*_ for the grain on the right hand side of the interface. The rotation of the Brillouin zone does not affect the aforementioned band structure alignment at the interface.

In 2D materials, GBs can be of different types depending on both the orientation of each grain with respect to the grain boundary and the orientation of the grains with respect to each other. One extreme is when both the grains are rotated symmetrically by equal angles away from the GB in opposite directions (i.e. Θ_*L*_ = Θ_*R*_ = Θ_*M*_/2) and the second is when only one of the grains is rotated away from the interface (i.e. Θ_*L*_ = 0°, Θ_*R*_ = Θ_*M*_). In literature, the former type of symmetric grain boundaries are referred to as twin GBs and the latter as the most asymmetric tilt GBs.

So far, we have discussed the two extreme cases of GBs for a given misorientation angle Θ_*M*_, but we can have many intermediate cases of tilt (asymmetric) GBs depending on the position of the boundary itself. For example, given that Θ_*M*_ is 4° we can have Θ_*L*_ = Θ_*R*_ = Θ_*M*_/2 (twin GBs), or Θ_*L*_ = 0° and Θ_*R*_ = Θ_*M*_ (the most asymmetric tilt GB), or intermediate cases such as Θ_*L*_ = 1° and Θ_*R*_ = 3°, and so on so forth. In order to denote these intermediate tilt cases, we introduce an angle Θ_*B*_, which is defined as the angle, in the anticlockwise direction, that the boundary makes with the reference line. So, an intermediate case of Θ_*L*_ = 1° and Θ_*R*_ = 3° can be represented as Θ_*M*_ = 4° and Θ_*B*_ = 1°.

The effect of GB/interface on transport is incorporated by using boundary conditions based on quantum-mechanical wave continuity^46^. From translational symmetry, transmission requires simultaneous conservation of energy and transverse momentum of the incident electron across the interface. Momentum conservation requires that the parallel component of the incident wave vector ${k}_{{i}_{\parallel }}$ be equal to the parallel component of the transmitted wave vector ${k}_{{t}_{\parallel }}$, in their respective domains that is ${k}_{{t}_{\parallel }}={k}_{{i}_{\parallel }}$; simultaneously, energy is conserved by finding a perpendicular component of the transmitted wave vector ${k}_{{t}_{\perp }}$, within the first Brillouin zone of the right grain, such that ${E}_{2}({k}_{{t}_{\parallel }}+{k}_{{t}_{\perp }})={E}_{1}({k}_{i})={E}_{1}({k}_{{i}_{\parallel }}+{k}_{{i}_{\perp }})$. Then we calculate the mode-dependent transmission coefficient ${\tau }_{b}(\overrightarrow{k})$ for each band *b* using the perpendicular components of the incident ${k}_{{i}_{\perp }}$ and transmitted ${k}_{{t}_{\perp }}$ wave vectors by the expression

1 $${\tau }_{b}({k}_{i})=\frac{|4{k}_{{i}_{\perp }}{k}_{{t}_{\perp }}|}{{|{k}_{{i}_{\perp }}+{k}_{{t}_{\perp }}|}^{2}}$$

Next, we obtain the energy-resolved transmission coefficient Γ_*b*_(*E*) by averaging the mode-dependent transmission coefficient *τ*_*b*_(*k*) over the constant energy contour, described by *δ*(*E* − *E*_*b*_(*k*)), using the 2D version of the linear extrapolation approach described by Gilat and Raubenheimer^47^ as

2 $${{\rm{\Gamma }}}_{b}(E)=\frac{\frac{1}{4{\pi }^{2}}\,\int \,{\tau }_{b}(k)\delta (E-{E}_{b}(k))dk}{\frac{1}{4{\pi }^{2}}\,\int \,\delta (E-{E}_{b}(k))dk}.$$

The denominator of Eq. 2 is the density of states in band *b* *D*_*b*_(*E*), shown in Fig. 1(b). The same transformation method is employed for converting the mode-dependent velocity ${v}_{{b}_{\parallel }}(k)$ into energy-resolved velocity ${v}_{{b}_{\parallel }}(E)$ in the direction of transport. We then calculate the transport distribution function TDF Ξ(*E*) as

3 $${\rm{\Xi }}(E)=\sum _{b}\,{v}_{{b}_{\parallel }}(E){{\rm{\Gamma }}}_{b}(E){D}_{b}(E)$$

The TDF is then used to numerically calculate the grain boundary conductance in the Landauer formalism and inverted to obtain the grain boundary resistance ${R}_{GB/int.}({G}_{GB/int.}^{-1})$. The grain boundary conductance is obtained from an integral of the product of TDF and Fermi window function ∂*f*(*E* − *E*_*F*_, *T*)/∂*E* over energy

4 $${G}_{GB/int.}=\frac{{e}^{2}}{2}\,{\int }_{{E}_{C}}^{{E}_{max}}\,{\rm{\Xi }}(E)(-\,\frac{\partial f(E-{E}_{F},T)}{\partial E})\,dE$$

where E_*C*_ is the bottom of the conduction band and E_*max*_ is the highest electron energy in the first four conduction bands and *f*(*E*) is the Fermi-Dirac distribution function *f*(*E*) = [1 + *exp*((*E* − *E*_*F*_)/*k*_*B*_ *T*)]^−1^.

## Results and Discussion

### Electron transport across graphene grain boundaries

**Figure 2:**
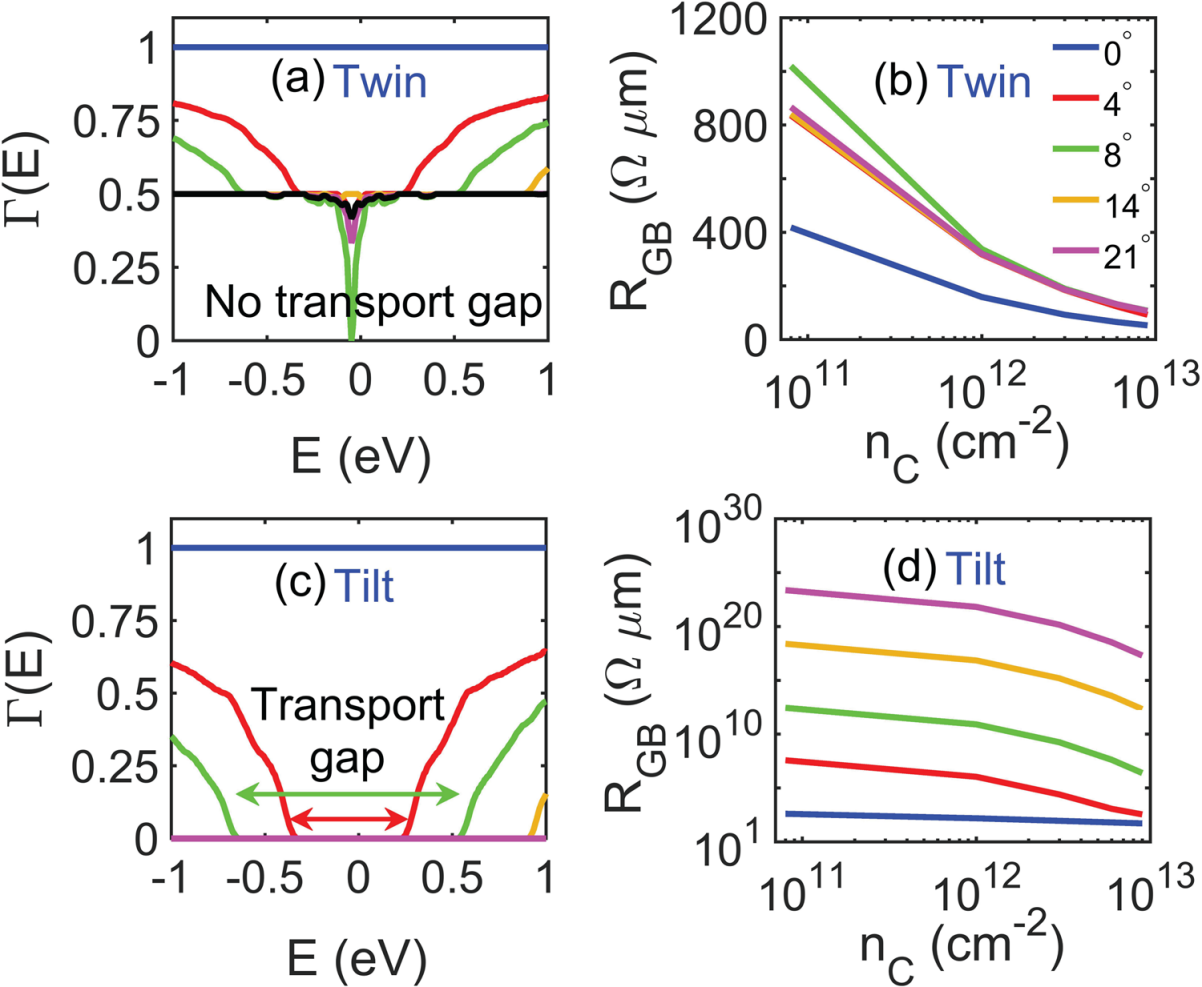
(**a**) Shows transmission coefficient vs. energy for various misorientation angles across graphene twin grain boundaries. (**b**) Shows the variation of grain boundary resistance with carrier concentration for the same mismatch angles as plotted in (**a**). The curves for large mismatch angles (14° and 21°) are overlapping on each other in both (**a**,**b**). Transmission coefficient vs. energy and the resultant GB resistance vs. carrier concentration for different misorientation angles in graphene tilt GBs are plotted in (**c**,**d**) respectively. A transmission gap opens up for tilt but not for twin GBs, resulting in much stronger angle dependence.

We calculate the transmission and resistance of graphene GBs in order to explore the impact of the misorientation angle. The angle dependence of GB resistance largely depends on the type (tilt or twin) of the GB. Figure 2(a,b) show transmission coefficient Γ(E) and GB resistance *R*_*GB*_ respectively for various misorientation angles in twin GBs. We see in Fig. 2(a) that perfect transmission, that is transmission coefficient Γ(*E*) equals 1, is obtained for 0° mismatch angle at any given energy level. However, as the misorientation angle increases, the modes that do not conserve energy and transverse momentum are reflected at the interface, resulting in a reduction of the transmission coefficient, which varies between 0.8 and 0.5 for various mismatch angles. Besides band gap, the energy range for which there is no transmission (Γ(E) = 0) is referred here as *transmission*/*transport gap*. In twin GBs, we note that even for large mismatch angles there is no transmission gap in the energy spectrum.

For GBs with 0° mismatch angle, we obtain a coefficient Γ(*E*) = 1; in contrast, Yazyev and Louie^15^ reported a linear transmission probability *T*(*E*) with energy. They used a non-equilibrium Green’s function (NEGF) formalism to calculate conductance across graphene grain boundaries. In the coherent transport regime, the conductance from NEGF formalism reduces to the conductance in Landauer formalism^48^, given by^49^ $G=\frac{{e}^{2}}{h}\,\int \,T(E)(-\,\frac{\partial f(E-{E}_{F},T)}{\partial E})dE$. Comparing this with our conductance expression (Eq. 4), we find that *T*(*E*) in the NEGF formailsm is analogous to our transport distribution function Ξ(*E*). For the graphene GBs with 0° mismatch, Ξ(*E*) in Eq. 3 is proportional to the DOS *D*_*b*_(*E*), which is linear with energy [as shown in Fig. 1(b) and Eq. 8]; thus our TDF is consistent with the *T*(*E*) vs. energy plot from NEGF^15^.

In Fig. 2(b), the GB resistance is plotted for different misorientation angles and carrier concentrations. For a given carrier concentration, the GB resistance increases with misorientation angles. This is due to the reduction in transmission coefficient with increasing misorientation angle, as can be seen in Fig. 2(a), which maps to an increase in GB resistance. Perfect transmission at 0° mismatch angle translates into ballistic resistance across graphene GBs as shown in Fig. 8(b) in the Appendix [same as the blue curve in Fig. 2(b,d)]. For a given mismatch angle, the GB resistance decreases with increasing carrier concentration as we can see in Fig. 2(b). At intrinsic carrier concentration, the Fermi level *E*_*F*_ is near the Dirac point in graphene. But with the increase in electron concentration the Fermi level goes into the conduction band, and consequently, the Fermi window function (−*df*/*dE*) which is centered at *E*_*F*_ also shifts towards higher energy levels. As the DOS in graphene is proportional to energy near the Dirac point (from Eq. 8 and Fig. 2(b)), the TDF Ξ(*E*) also increases with energy away from the Dirac point. Thus the value of the integral in Eq. 4, which is a product of TDF and Fermi window function, increases with carrier concentration. As a result, we see a decrease in GB resistance with increasing carrier concentration in Fig. 2(b).

Figure 2(c,d) show transmission coefficient Γ(E) and grain boundary resistance respectively for various mismatch angles in tilt grain boundaries. The transmission coefficient shows a similar reduction with increasing mismatch angles as seen in Fig. 2(a); however the reduction is more pronounced than in the case of twin GBs. In tilt GBs we also observe a widening of the transmission gap, shown in the Fig. 2(c), with increasing misorientation angle. This transmission gap around the Dirac point maps into large GB resistance for large-angle tilt GBs and grain boundary resistance becomes less sensitive to the variation in carrier concentration.

Previously, it was found that the GB resistance across graphene GBs varies within a wide range from a few Ω *μ*m to several kΩ *μ*m^2,3^. The wide variation in GB resistance can be fully explained with the trends observed in Fig. 2(b,d): there is a large difference in resistance between twin and tilt GBs, with twin GBs being less sensitive to misorientation angles as compared to the tilt GBs. The GB resistance in tilt GBs range from about 350 Ω *μ*m at 4° mismatch to several thousands of GΩ *μ*m at 14° mismatch, even at high carrier concentration of about 10^13^ cm^−2^. The transmission coefficient eventually becomes zero for misorientation angles beyond 14° mismatch due to the large transmission gap in tilt GBs and we observe extremely high values of resistances. In contrast, the resistance of twin GBs in near-intrinsic graphene varies from 400 Ω *μ*m at low to about 1 kΩ *μ*m at high mismatch angles, while at high carrier concentration it varies over a very narrow range of about 90 Ω *μ*m at 4° mismatch to 110 Ω *μ*m at 14° mismatch. Our calculated graphene GB resistances include a ballistic resistance of 53 Ω *μ*m at high carrier concentration of 10^13^ cm^−2^ and 424 Ω *μ*m at intrinsic carrier concentration. After removing the ballistic resistance, the calculated GB resistance for a low-mismatch twin GBs of about 1° at high carrier concentration is 8 Ω *μ*m. This is in good agreement with Grosse *et al*.^11^.

### Electron transport across MoS_2_ grain boundaries

**Figure 3:**
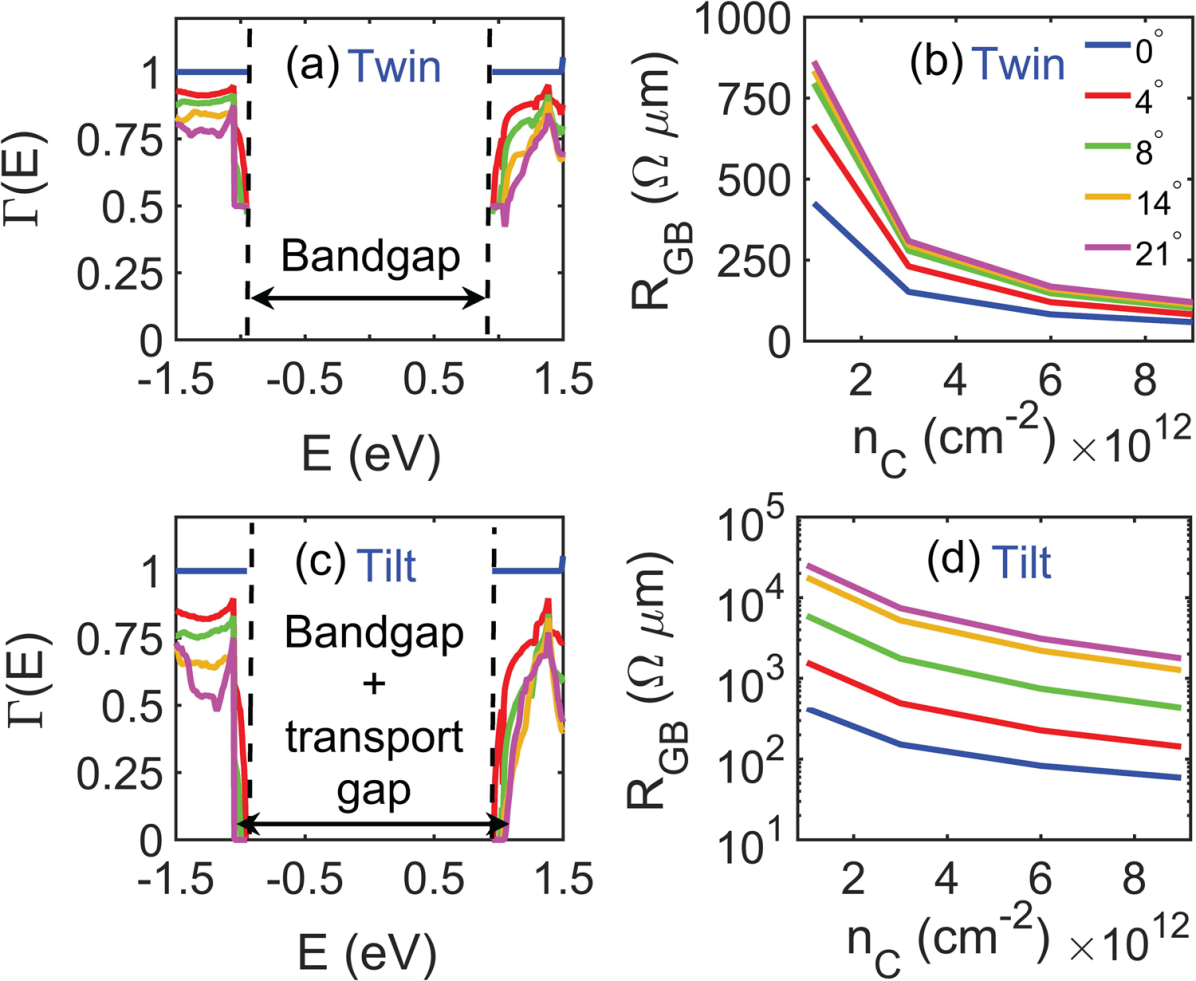
(**a**) Shows transmission coefficient vs. energy for various misorientation angles across MoS_2_ twin grain boundaries. (**b**) Shows the variation of grain boundary resistance with carrier concentration for the same mismatch angles as plotted in (**a**). Transmission coefficient vs. energy and the resultant GB resistance vs. carrier concentration for different misorientation angles in MoS_2_ tilt GBs are plotted in (**c**,**d**) respectively. Besides intrinsic band gap, an additional transmission gap opens up for large tilt GBs.

To study electronic resistance across MoS_2_ GBs, we use the same procedure as used for graphene GBs in the previous section. The transmission coefficient as a function of energy is plotted in Fig. 3(a) for different misorientation angles in twin GBs. The blue curve shows transmission across an imaginary, perfectly-matched grain boundary (which corresponds to 0° mismatch). A perfect transmission is obtained for energies greater than about 0.94 eV and less than about −0.94 eV. Zero transmission at energies between −0.94 eV and 0.94 eV corresponds to the energy band gap of 1.88 eV in intrinsic MoS_2_. We also observe a gradual reduction in transmission coefficient with increasing misorientation angles as compared to that of graphene GBs. However, there is no transmission gap found for MoS_2_ twin GBs, similar to what we observed in graphene twin GBs. Corresponding to the transmission coefficient for various misorientation angles, the boundary resistance across MoS_2_ twin grain boundaries vs. carrier concentration is shown in Fig. 3(b). We note that the values of *R*_*GB*_ in MoS_2_ twin GBs are almost double than the values of GB resistance in graphene twin boundaries for a carrier concentration of 1 × 10^12^ cm^−2^, whereas for large carrier concentrations between 6 × 10^12^ and 9 × 10^12^ cm^−2^, MoS_2_ twin GBs have GB resistance similar to that of graphene twin GBs.

Figure 3(c,d) show transmission coefficient vs. energy and GB resistance vs. carrier concentration respectively for various misorientation angles in MoS_2_ tilt GBs. It can be seen in Fig. 3(c) that the transmission coefficient decreases with increasing misorientation angle and the rate of reduction of transmission coefficient is more rapid than what was observed in MoS_2_ twin GBs. Like in tilt graphene GBs, a transmission gap is also observed in tilt MoS_2_ GBs for large misorientation angles, however the transmission gap in MoS_2_ is much smaller than that of graphene. We attribute this trend to the flatter parabolic conduction band bottom of MoS_2_ as compared to the steep conical bandstructure of graphene around the Dirac point. The variation of GB resistance with misorientation angle is quite distinct in this case as compared to the variation of *R*_*GB*_ in graphene tilt GBs. It is important to note that the resistance across MoS_2_ GBs is much smaller than what we found in graphene tilt GBs. Thus, misorientation of adjacent grains across grain boundaries can cause a significant reduction in electronic conductance in polycrystalline graphene, while GBs in polycrystalline MoS_2_ might not play such a strong role in electron conduction, which is in good agreement with few recent reports on electronic transport in CVD-grown MoS_2_^21,22^.

**Figure 4:**
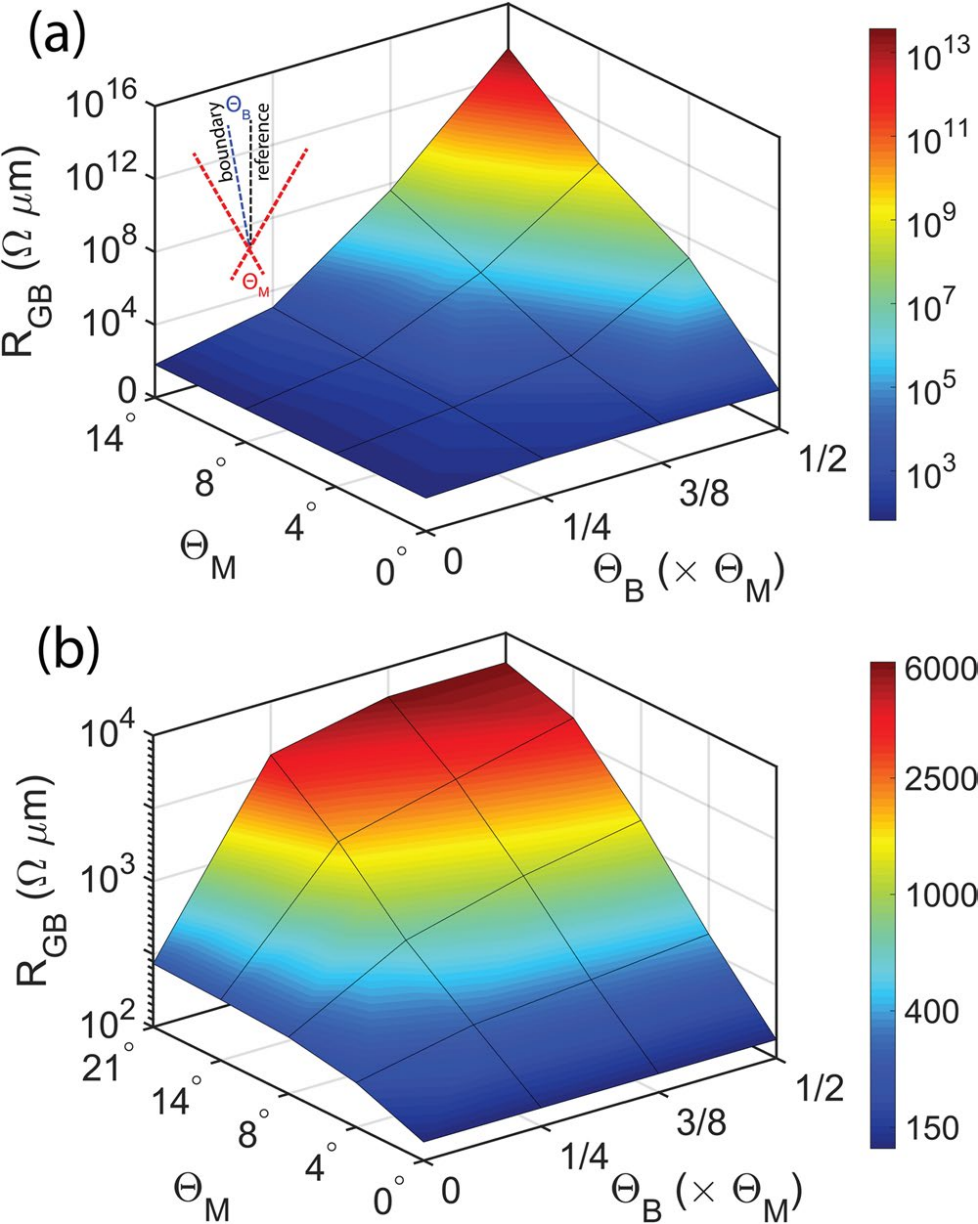
Shows resistance (**a**) across graphene GBs and (**b**) across MoS_2_ GBs vs. misorientation angles Θ_*M*_ and various combinations of Θ_*L*_ and Θ_*R*_, represented as Θ_*B*_ for a given Θ_*M*_. Here Θ_*B*_ is expressed as a fraction of Θ_*M*_.

Figure 4(a,b) depict the surface plots of GB resistance vs. Θ_*M*_ and Θ_*B*_ for graphene and MoS_2_ GBs respectively. The calculated value of resistances across graphene GBs range from few tens of Ω *μ*m to about 10^13^ Ω *μ*m depending on the Θ_*M*_ (the angle between the two grains) and Θ_*B*_ (the position of the boundary with respect to the left grain). However, the GB resistance across MoS_2_ GBs vary over a relatively narrow range of about 130 Ω *μ*m to 5700 Ω *μ*m for various combinations of Θ_*M*_ and Θ_*B*_. Thus, we can see that for a given misorientation angle, one can have different GB resistance depending on the position of the GB with respect to the grains, and any resistance value falling in this range can be explained by a combination Θ_*M*_ and Θ_*B*_.

### Electron transport across graphene-MoS_2_ interfaces

The interfaces formed between two dissimilar materials (heterojunctions) are different from those of homojunctions because of the difference in the properties of the grains on either side of the interface—including electron affinity, work function, and bandstructure. So, before discussing about electron transport across such heterojunctions, we redefine the nomenclature of the interfaces formed between graphene and MoS_2_ to differentiate with those of homojunctions. When graphene (taken here to be on left side of the boundary) and MoS_2_ (right side of the boundary) grains are rotated by equal angles with respect to the interface i.e. Θ_*L*_ = Θ_*R*_, we use the term Class-I interface, whereas when ${{\rm{\Theta }}}_{L}\ne {{\rm{\Theta }}}_{R}$ we call them Class-II interfaces in this work.

**Figure 5:**
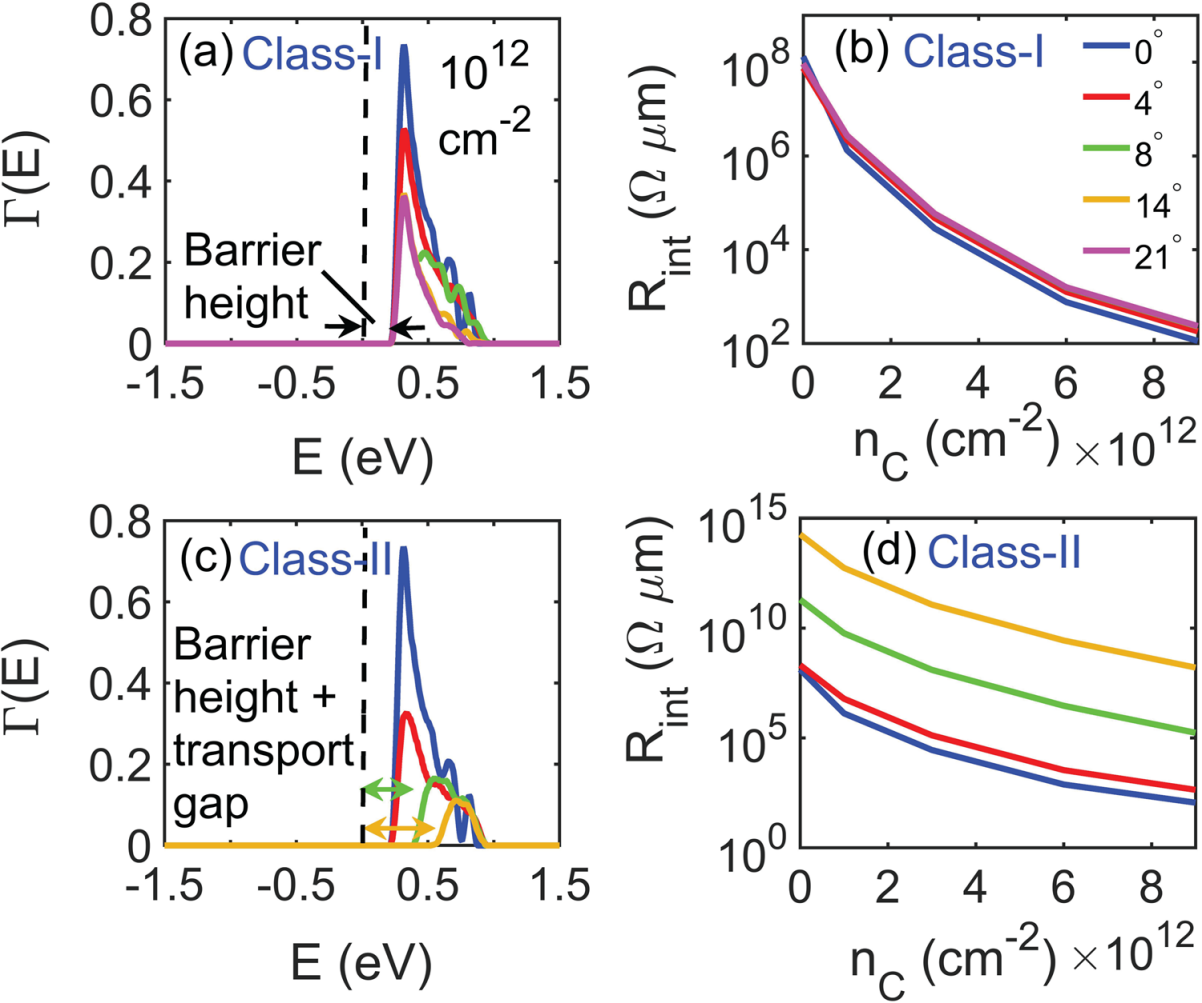
(**a**) Shows transmission coefficient vs. energy for various misorientation angles across graphene-MoS_2_ Class-I interfaces. (**b**) Shows the variation of interface resistance with carrier concentration for the same mismatch angles as plotted in (**a**). Class-I graphene-MoS_2_ interfaces show neglibible sensitivity towards misorientation angles. (**c**) Shows transmission coefficient vs. energy for different misorientation angles in graphene-MoS_2_ Class-II interfaces. On top of intrinsic barrier height, an additional transmission gap opens up for such Class-II graphene-MoS_2_ interfaces. The resulting interface resistance in Class-II interfaces vs. carrier concentration for different misorientation angles are plotted in (**d**).

Figure 5(a) shows the thermionic transmission of the electrons across graphene-MoS_2_ Class-I interface for various misorientation angles at a carrier concentration of 1 × 10^12^ cm^−2^. Due to the difference in the work function and electron affinity in graphene and MoS_2_, the bands bend and an energy barrier (the energy difference between fermi-level, approximately equal to 0 eV in Fig. 5(a,c), to the bottom of the conduction band of MoS_2_ at the interface where the transmission of electrons start) is formed at the interface. Like in homojunctions, the band alignment at the interface is independent of the misorientation angle, and thus, the barrier height is also independent of Θ_*M*_. In Fig. 5(b), we see that at low carrier concentrations of about 1 × 10^12^ cm^−2^, the interface resistance is in the order of 10^8^ Ω *μ*m because of the large energy barrier. However, at high carrier concentrations between 6 × 10^12^ cm^−2^ and 9 × 10^12^ cm^−2^, the interface resistance reduces significantly because the energy barrier between graphene and MoS_2_ almost disappears at such concentrations and they behave like Ohmic contacts.

Figure 5(c) shows transmission coefficient vs. energy for various misorientation angles in Class-II graphene-MoS_2_ interfaces at a carrier concentration of 1 × 10^12^ cm^−2^. The transmission coefficient decreases with increasing mismatch angle similar to the tilt GBs in graphene GBs. A transmission gap is formed in addition to the existing potential barrier, marked in the figure, and this transmission gap widens with increasing misorientation angles. Transmission becomes zero for large mismatch angles beyond 14°. This strong dependence of mismatch angle on transmission coefficient leads to a strong dependence of the interface resistance on misorientation angles in Class-II graphene-MoS_2_ heterojunctions, which can be seen in Fig. 5(d). At a carrier concentration of 1 × 10^12^ cm^−2^, the interface resistance varies from about 10^8^ for low mismatch angles to 10^14^ Ω *μ*m for a mismatch of 14°, whereas at high concentrations the interface resistance ranges from about 10^2^ for low mismatch angles to 10^8^ Ω *μ*m for a mismatch of 14°.

**Figure 6:**
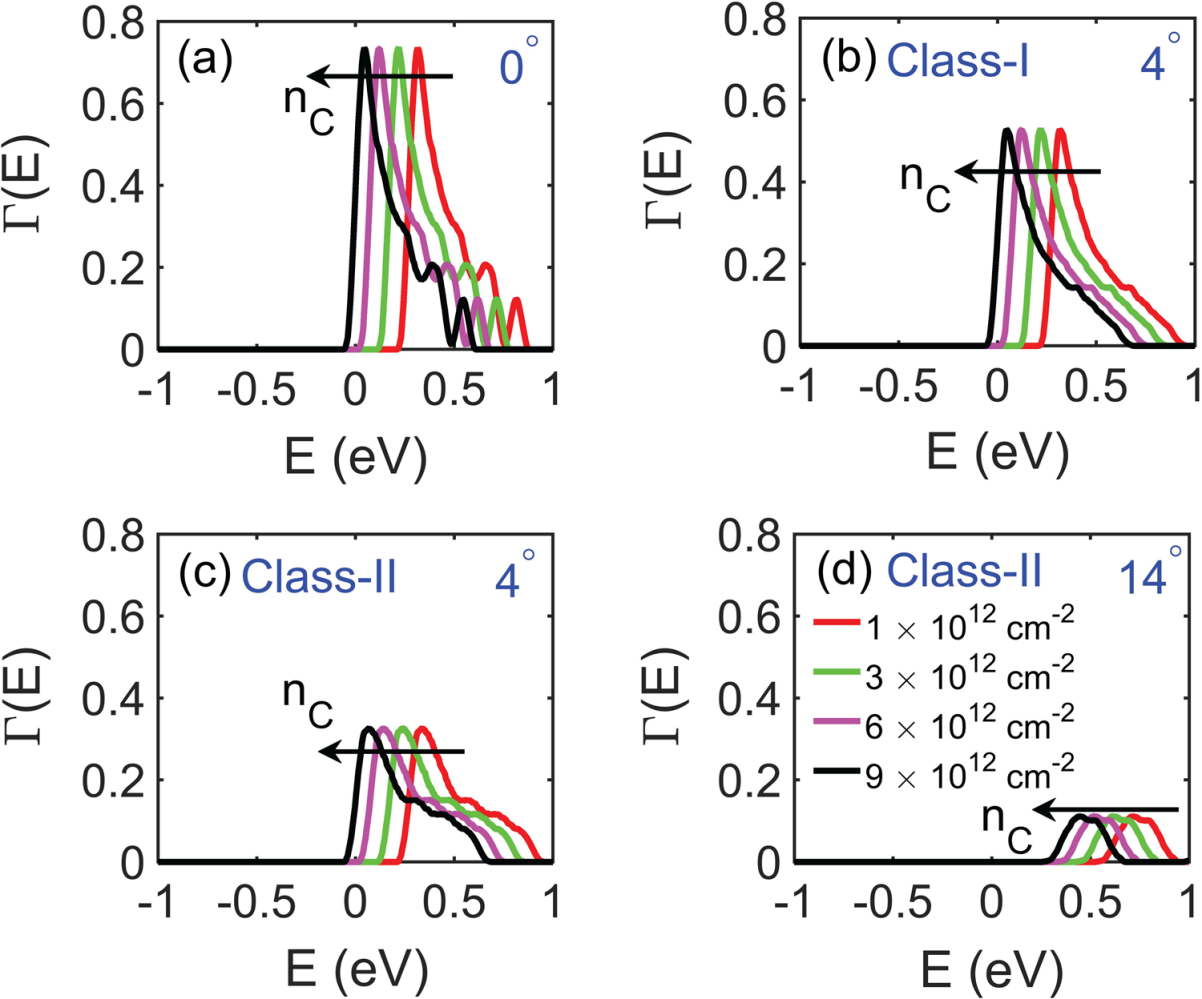
Show transmission coefficient vs. energy for various carrier densities (**a**) for 0° mismatch, (**b**,**c**) for 4° in Class-I and Class-II graphene-MoS_2_ interfaces respectively, and (**d**) for 14° mismatch in Class II interfaces.

In homojunctions like graphene-graphene and MoS_2_-MoS_2_ GBs, the band alignment is independent of the position of the Fermi level so the transmission coefficient is also independent of carrier concentration. In contrast, the barrier height in heterojunctions is a function of carrier concentration via the position of the Fermi level, owing to the difference in DOS between graphene and MoS_2_. The transmission coefficient in Class-I graphene-MoS_2_ interface is plotted with carrier concentration in Fig. 6. Figure 6(a) shows the transmission coefficient vs. energy for perfectly matched graphene-MoS_2_ interface, that is 0° mismatch. The shape of the Γ(E) vs. energy does not change with carrier concentration rather the curves get shifted towards the left in energy due to the decrease in energy barrier height with carrier concentration. In Fig. 6(b,c), the transmission coefficient vs. energy is plotted for 4° mismatch in Class I and II heterojunctions respectively. The decrease in the magnitude of transmission coefficient as compared to that of Fig. 6(a) is more pronounced for Class II than Class-I interfaces. For large misorientation angles, that is beyond 14° the transmission coefficient in Class II interfaces becomes very small about 0.1 eV as can be seen in Fig. 6(d), whereas for Class I heterojunctions the transmission coefficient peak is about 0.4 eV (shown by the green curve in Fig. 5(a), noting that a change in carrier concentration only shifts the Γ vs E curve and does not change the shape).

**Figure 7:**
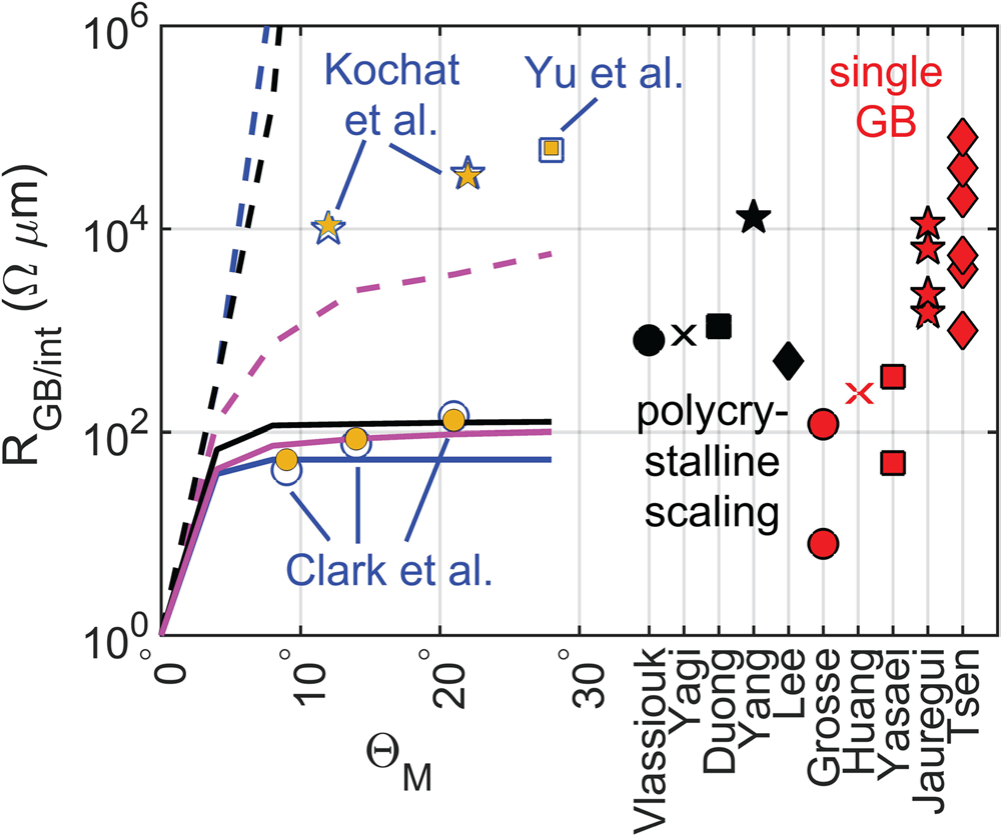
Compares the calculated GB/interface resistance vs. misorientation angles across twin (solid lines) and tilt (dashed lines) Gr-Gr (shown in blue) and MoS_2_-MoS_2_ (shown in magenta) GBs, and Class-I (solid black line) and Class-II (dashed black line) Gr-MoS_2_ interfaces. The reported values of graphene GB resistance in the literature are also plotted in this figure. The data for graphene GB resistance which are available with misorientation angles are plotted with blue markers^3,14,50^. The other studies about graphene GB resistance where mismatch angle wasn’t mentioned explicitly are plotted on the right with red and black markers. The red markers are the resistance values from literature across single graphene GB^2,4,11,12,60^. The black markers are for the literature data on GB resistance extracted by polycrystalline scaling from polycrystalline samples^61–65^. The yellow markers represent the calculated graphene GB resistance corresponding to those combinations of Θ_*M*_ and Θ_*B*_ which fit the experimental measurements.

Figure 7 shows a comparison of the interface resistance among Gr-Gr, MoS_2_-MoS_2_, and Gr-MoS_2_ interfaces. It can be seen that, in general, symmetric twin GBs in homojunctions and Class-I interfaces in heterojunctions show a very weak dependence on the degree of mismatch between adjacent grains, whereas tilt GBs in homojunctions and Class-II interfaces in heterojunctions exhibit strong dependence on misorientation angles except in MoS_2_, where both tilt and twin GBs are found to show a weak dependence on mismatch angles. The weak angle dependence in MoS_2_-MoS_2_ GBs can be attributed to the flat parabolic conduction band because of which the underlap in the bandstructures on the either side of the GB is quite small even at large mismatch angles. In order to explain the wide range of the graphene GB resistances in the literature via misorientation angle and type of GBs, the data from the literature has also been included in the figure. The yellow markers in the figure denote the combinations of Θ_*M*_ and Θ_*B*_ obtained by fitting the experimental measurements by Kochat *et al*.^50^ (Θ_*B*_ = 2.83° and 3° for Θ_*M*_ = 12° and 22° respectively), Clark *et al*.^14^ (Θ_*B*_ = 0°, 0.2° and 0.75° for Θ_*M*_ = 9°, 14°, and 21° respectively), and Yu *et al*.^3^ (Θ_*B*_ = 3.1° for Θ_*M*_ = 28°).

The aim of our study is to understand the effect of misorientation angle on GB conductance. There could be additional effects due to grain boundary roughness^9^ and the presence of localized electronic states, which have been observed at 3D interfaces^51^ and 2D grain boundaries^25,52,53^. The localized states are not included in our model, but could be treated by modifying *D*_*b*_(*E*) in Eq. 3. The presence of localized interface states could lead to two types of behavior, depending on the magnitude of the transport gap. For low mismatch angles or symmetric GBs where the transport gap is small and the transmission coefficient is close to unity, the localized states and roughness at the GB would reduce the transmission coefficient; in that case, our calculated conductance values can be thought of as an upper bound. For large mismatch angles, we found a wide transport gap where transmission is zero, especially in graphene GBs and graphene-MoS_2_ interfaces. Then localized states might introduce additional channels for transmission and lead to slightly higher GB conductance than what we report here without these localized states. In that sense, our conductance values could be thought of as a lower bound.

## Conclusion

In conclusion, we find that misorientation angle between two adjacent grains plays a very significant role in both homojunctions and heterojunctions. We show that the resistance across graphene GBs and graphene-MoS_2_ interfaces varies over a very wide range depending on the degree of mismatch between adjacent grains and type of GBs. The transmission coefficient across symmetric interfaces (twin GBs in homojunctions and Class-I interfaces in heterojunctions) is found to be less sensitive to misorientation angles between adjacent grains because they deflect electrons rather coherently. In these symmetric interfaces, there is no transmission gap. On the other hand, the transmission across asymmetric interfaces (tilt GBs in homojunctions and Class-II interfaces in heterojunctions) is largely diminished by mismatch angles and a transmission gap opens up in the energy spectrum. In contrast to graphene-graphene tilt GBs, the resistance across MoS_2_-MoS_2_ tilt GBs show relatively much weaker dependence on mismatch angles. This is attributed to the flat parabolic conduction band bottom in MoS_2_ as compared to the steep conical conduction band bottom in graphene. As a result, the rotation of the BZ in MoS_2_ by large angles causes a small transmission gap, whereas even a small misorientation angle across graphene GBs gives rise to a large transmission gap. In homojunctions, the bands are identical on either side of the interface and the response to the carrier concentration, and hence the back-gated voltage, is also uniform on both sides. Thus, for a given misorientation angle, the variation of transmission coefficient vs. energy is independent of carrier concentration in homojunctions. In heterojunctions, the bands are aligned at the interface using the macroscopic variables, including work function and electron affinity, based on Schottky-Mott rule, forming an energy barrier at the interface. The band alignment, and with it the energy barrier between graphene and MoS_2_, reduces with the carrier concentration because of the differences in their densities-of-states. Consequently, the interface resistance strongly decreases with carrier concentration in heterojunctions in both classes of interfaces. In summary, electrical transport across twin homojunctions and Class-I heterojunctions shows a weak dependence on mismatch angles, whereas the resistance across tilt homojunctions and Class-II heterojunctions exhibits a strong dependence on mismatch angles.

## Methods

### Density Functional Theory calculations of the electronic bandstructure

For graphene, we used a scalar relativistic, norm-conserving pseudopotential (NCPP) which implements a direct-fit Von Barth-Car method with a Perdew-Zunger local density approximation (LDA) exchange-correlation functional^54^. For MoS_2_, we used a nonrelativistic NCPP for Mo and a scalar relativistic NCPP for S. Both potentials employed a Martins-Troullier method with a Perdew-Wang LDA exchange correlation^55^. The lattice constants are a = 2.459 Å for graphene and a = 3.125 Å, z = 3.11 Å for MoS_2_, where z is the distance between chalcogen atoms. To ensure that interplanar interactions are neglected, the repeating images of the monolayers are seperated by a 20 Å vacuum. The cutoff energy for plane waves was 120 Ry for graphene and 140 Ry for MoS_2_. We used a convergence threshold of 10^−15^ on a Monkhorst-Pack grid sizes of 8 × 8 × 1 for graphene and 6 × 6 × 4 for MoS_2_ for the initial total energy calculation and then performed a bandstructure calculation on a dense grid of 25,208 k-points (wavevectors) with a convergence threshold of 10^−12^. We used the central difference method to obtain the electron velocities per band which, in turn, are subsequently used in calculating the electronic density of states (DOS) and other transport properties including interfacial transmission and resistance of the interface.

### Appendix: Ballistic resistance of graphene-graphene interface

We derive an analytical expression for the ballistic resistance across graphene GBs and compare it with the numerically computed values of ballistic resistance at different carrier concentrations. We define ballistic resistance as the resistance between two perfectly-matched grains, that is when misorientation angle (Θ_*M*_) is 0°. In the diffusive limit (Ohmic regime) when the dimension of the conductor is large compared to the carrier mean free path, the conductance varies inversely with length. One would expect the conductance to become infinite when the conductor length tends to zero. However, it has been experimentally found in both metals^56^ and semiconductors^57,58^ that the measured conductance converge to a finite value called ballistic conductance. The regime where we see this limiting behavior is called ballistic regime. In this regime, characterized by an absence of scattering, the conductor has no resistance–the ballistic resistance is not the resistance of the conductor but the contact resistance^48^.

With a careful treatment of the voltage across the GB, as done in a 4-probe measurement and analogous to the corrections made to the temperature gradient for phonon transmission^46^, one could remove the ballistic contact resistance and show that the resistance across an idealized perfectly-matched GB is zero. In our calculations, the GB resistance at any given mismatch angle includes the ballistic resistance, which varies with the carrier concentration. So, while comparing the 4-probe experimental measurements of GB resistance for a given mismatch angle with our results, as shown in Fig. 7, we subtract the ballistic resistance from the calculated GB resistance.

The ballistic conductance for 1D conductor is given by the expression *G*_*ball*,1*D*_ = 2*e* ^2^/*h*, where *e* is the charge of the carrier and *h* is Planck’s constant. A 2D conductor of width *W* could be thought of as a number of parallel 1D conductors, and thus, the conductance of the 2D conductor is the sum of the conductances of all the 1D conductors. The number of such 1D conductors that would be equivalent to the 2D conductor of width *W* is called 2D channel number, *M*_2*D*_. Using the expression of ballistic conductance for 1D conductor, we write the ballistic conductance for a two-dimensional conductor as

5 $${G}_{ball\mathrm{,2}D}={G}_{ball\mathrm{,1}D}\times {M}_{2D}({E}_{F})$$

The channel number at any energy (E) for a given width of the ribbon is calculated as^59^

6 $$M(E)=W\frac{h}{4}\langle {v}_{x}(E)\rangle {D}_{2D}(E)$$

where 〈*v*_*x*_(*E*)〉 is calculated by 2D averaging of velocity of all the modes, $\langle {v}_{x}(E)\rangle =\frac{2}{\pi }{v}_{F}$. *v*_*F*_ is the Fermi velocity (≈10^6^ ms^−1^), which is computed from the slope of the bandstructure (E-k relationship) around Dirac point. *D*_2*D*_(*E*) is the 2D density of states.

The dispersion of graphene around the Dirac point is approximated by the relation $E(\vec{k})=\hslash {v}_{F}|\vec{k}|$, where $\hslash$ is the reduced Planck’s constant. The general expression for calculating 2D density of states is

7 $${D}_{2D}(\vec{k})=\frac{1}{{\mathrm{(2}\pi )}^{2}}\frac{2\pi |\vec{k}|}{{\nabla }_{k}E(\vec{k})}{g}_{s}{g}_{v}$$

where *g*_*s*_ and *g*_*v*_ are constants related to the spin of electron and valley degeneracy respectively. For graphene *g*_*v*_ = 2 and *g*_*s*_ = 2 for electrons. ${\nabla }_{k}E(\vec{k})$ is the gradient of energy dispersion with respect to the wavevector and around the Dirac point it can be approximated by $\hslash {v}_{F}$. Thus for graphene,

8 $${D}_{2D}(E)=\frac{2}{\pi {\hslash }^{2}{v}_{F}^{2}}|E|$$

In general, 2D carrier concentration is given as

9 $${n}_{2D}({E}_{F})={\int }_{0}^{\infty }\,{f}_{0}(E){D}_{2D}(E)dE$$

where *f*_0_(*E*) is the equilibrium Fermi-Dirac distribution function, ${f}_{0}(E)={[1+exp(\frac{E-{E}_{F}}{{K}_{B}T})]}^{-1}$. As graphene is degenerate, so Eq. 9 can be approximated by

10 $${n}_{2D}({E}_{F})={\int }_{0}^{{E}_{F}}\,{D}_{2D}(E)dE={\int }_{0}^{{E}_{F}}\,\frac{2}{\pi {\hslash }^{2}{v}_{F}^{2}}EdE=\frac{{E}_{F}^{2}}{\pi {\hslash }^{2}{v}_{F}^{2}}$$

Using the expressions for density of states and 2D-averaged velocity, channel number in Eq. 6 for graphene can be written as

11 $${M}_{2D}({E}_{F})=\frac{2}{\pi }\frac{{E}_{F}}{\hslash {v}_{F}}W$$

Replacing the expression for channel number obtained from Eq. 11 in Eq. 5, we can calculate ballistic conductance in graphene as

12 $$\frac{{G}_{ball\mathrm{,2}D}}{W}=\frac{8{q}^{2}}{{\hslash }^{2}{v}_{F}}{E}_{F}$$

From Eqs 10 and 12, ballistic conductance in graphene can be expressed in terms of carrier concentration as

13 $$\frac{{G}_{ball\mathrm{,2}D}}{W}=\frac{4{q}^{2}}{h}\sqrt{\frac{{n}_{2D}}{\pi }}$$

Thus the ballistic resistance *R*_*ball*_ in graphene, which is the reciprocal of *G*_*ball*,2*D*_, is inversely proportional to the square root of carrier concentration. On using the values of the constants in Eq. 13 and intrinsic carrier concentration of 8 × 10^10^ cm^−2^, the analytical value of *R*_*ball*_ in graphene is about 405 Ω *μ*m.

**Figure 8:**
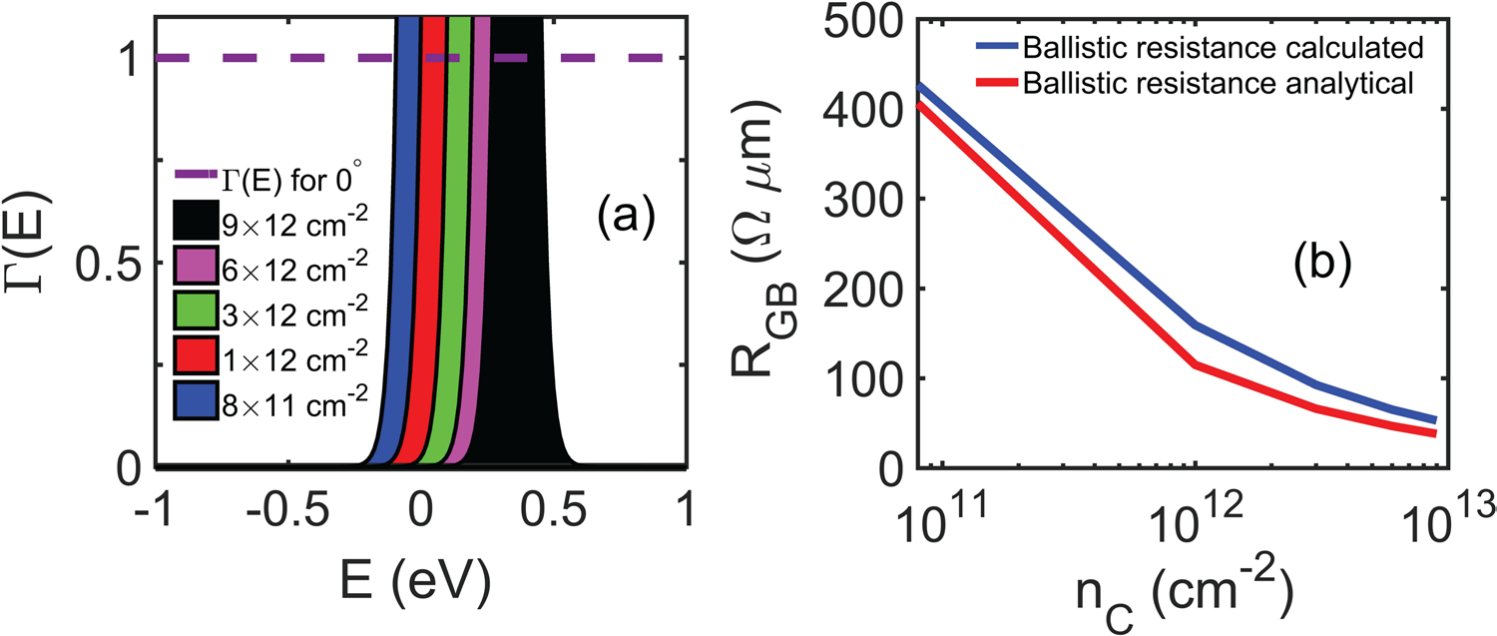
(**a**) Shows perfect transmission for 0° mismatch angle between two graphene grains. The curves outlining the area in different colors represent the Fermi window function (−*df*/*dE*), which is symmetric about *E*_*F*_, for different carrier concentrations. (**b**) Shows comparison between numerically and analytically calculated values of GB resistance (*R*_*GB*_) with carrier concentration. *R*_*GB*_ is inversely proportional to the square root of the carrier concentration.

We compare these analytically calculated values of ballistic resistance for different carrier concentrations with our numerically calculated values as shown in Fig. 8(b). At intrinsic carrier concentration (*n*_0_ = 8 × 10^10^ cm^−2^), the numerically calculated ballistic resistance is 424 Ω *μ*m, which compares quite well with the analytical value of 405 Ω *μ*m. In Fig. 8(a) we can see that transmission coefficient Γ(*E*) is 1 for the entire energy range, showing perfect transmission for 0° mismatch (perfectly-matched grains). The curve encompassing the blue area in the figure is the Fermi window function, which is defined as the derivative of Fermi-Dirac distribution function with respect to energy, for intrinsic carrier concentration that is when the Fermi level *E*_*F*_ is around the Dirac point and the electron concentration is equal to the hole concentration. Eq. 4 shows that the grain boundary resistance is a function of transmission coefficient, Fermi window function, velocity (proportional to Fermi velocity in graphene, which is a constant) and density of states. When the carrier (electron) concentration increases the Fermi level goes inside the conduction band and as a result Fermi window function, which is symmetric about the Fermi level, also shifts accordingly as shown in Fig. 8(a). The integral of the product of Fermi window, transmission coefficient and velocity with respect to the energy is the same for all carrier concentrations but it is due to the 2D density of states, which is independent of the Fermi level, in the integral of grain boundary resistance that causes the difference in *R*_*GB*_ when plotted against carrier concentration as shown in Fig. 8(b).

### Data Availability

 The datasets generated during and/or analysed during the current study are available from the corresponding author on reasonable request.

## References

[1] Li X (2010). Graphene films with large domain size by a two-step chemical vapor deposition process. Nano Letters.

[2] Tsen AW (2012). Tailoring electrical transport across grain boundaries in polycrystalline graphene. Science.

[3] Yu Q (2011). Control and characterization of individual grains and grain boundaries in graphene grown by chemical vapour deposition. Nat. Mater..

[4] Jauregui LA, Cao H, Wu W, Yu Q, Chen YP (2011). Electronic properties of grains and grain boundaries in graphene grown by chemical vapor deposition. Solid State Communications.

[5] Mataré HF (1984). Carrier transport at grain boundaries in semiconductors. Journal of Applied Physics.

[6] Isacsson A (2017). Scaling properties of polycrystalline graphene: a review. 2D Materials.

[7] Ma T (2017). Tailoring the thermal and electrical transport properties of graphene films by grain size engineering. Nat. Commun..

[8] Mortazavi B (2017). Strong thermal transport along polycrystalline transition metal dichalcogenides revealed by multiscale modeling for mos_2_. Applied Materials Today.

[9] Yasaei P (2015). Bimodal phonon scattering in graphene grain boundaries. Nano Lett..

[10] Aksamija Z, Knezevic I (2014). Lattice thermal transport in large-area polycrystalline graphene. Phys. Rev. B.

[11] Grosse KL (2014). Direct observation of resistive heating at graphene wrinkles and grain boundaries. Appl. Phys. Lett..

[12] Huang PY (2011). Grains and grain boundaries in single-layer graphene atomic patchwork quilts. Nature.

[13] Koepke JC (2013). Atomic-scale evidence for potential barriers and strong carrier scattering at graphene grain boundaries: A scanning tunneling microscopy study. ACS Nano.

[14] Clark KW (2013). Spatially resolved mapping of electrical conductivity across individual domain (grain) boundaries in graphene. ACS Nano.

[15] Yazyev OV, Louie SG (2010). Electronic transport in polycrystalline graphene. Nat. Mater..

[16] Vancsó P (2013). Electronic transport through ordered and disordered graphene grain boundaries. Carbon.

[17] Zhang H, Lee G, Gong C, Colombo L, Cho K (2014). Grain boundary effect on electrical transport properties of graphene. J. Phys. Chem. C.

[18] Sun J (2016). Electronic and transport properties of graphene with grain boundaries. RSC Adv..

[19] Ophus C, Shekhawat A, Rasool H, Zettl A (2015). Large-scale experimental and theoretical study of graphene grain boundary structures. Phys. Rev. B.

[20] Najmaei S (2014). Electrical transport properties of polycrystalline monolayer molybdenum disulfide. ACS Nano.

[21] Kang K (2015). High-mobility three-atom-thick semiconducting films with wafer-scale homogeneity. Nature.

[22] Schmidt H (2014). Transport properties of monolayer mos_2_ grown by chemical vapor deposition. Nano Letters.

[23] van der Zande AM (2013). Grains and grain boundaries in highly crystalline monolayer molybdenum disulphide. Nat. Mater..

[24] Najmaei S (2013). Vapour phase growth and grain boundary structure of molybdenum disulphide atomic layers. Nat. Mater..

[25] Ly TH (2016). Misorientation-angle-dependent electrical transport across molybdenum disulfide grain boundaries. Nat. Commun..

[26] Behranginia, A. *et al*. Direct growth of high mobility and low-noise lateral mos_2_ graphene heterostructure electronics. *Small* 1604301– (2017). 10.1002/smll.201604301 28626881

[27] Ci L (2010). Atomic layers of hybridized boron nitride and graphene domains. Nat. Mater..

[28] Han GH (2013). Continuous growth of hexagonal graphene and boron nitride in-plane heterostructures by atmospheric pressure chemical vapor deposition. ACS Nano.

[29] Liu Z (2013). In-plane heterostructures of graphene and hexagonal boron nitride with controlled domain sizes. Nat. Nano..

[30] Deng Y (2014). Black phosphorus–monolayer mos_2_ van der waals heterojunction p–n diode. ACS Nano.

[31] Stradi D, Papior NR, Hansen O, Brandbyge M (2017). Field effect in graphene-based van der waals heterostructures: Stacking sequence matters. Nano Lett..

[32] Kistanov AA, Cai Y, Zhang Y-W, Dmitriev SV, Zhou K (2017). Strain and water effects on the electronic structure and chemical activity of in-plane graphene/silicene heterostructure. J. Phys. Condens. Matter.

[33] Yu L (2014). Graphene/mos_2_ hybrid technology for large-scale two-dimensional electronics. Nano Lett..

[34] Yu H, Kutana A, Yakobson BI (2016). Carrier delocalization in two-dimensional coplanar p–n junctions of graphene and metal dichalcogenides. Nano Lett..

[35] Tian, H. *et al*. Novel field-effect schottky barrier transistors based on graphene-mos_2_ heterojunctions. *Sci*. *Rep*. **4**, 5951– (2014). 10.1038/srep05951 PMC4127518 25109609

[36] Liu X, Li Z (2015). Electric field and strain effect on graphene-mos_2_ hybrid structure: Ab initio calculations. J. Phys. Chem. Lett..

[37] Logoteta, D., Fiori, G. & Iannaccone, G. Graphene-based lateral heterostructure transistors exhibit better intrinsic performance than graphene-based vertical transistors as post-cmos devices. *Sci*. *Rep* **4**, 6607 (2014). 10.1038/srep06607 PMC4202216 25328156

[38] Giannozzi P (2009). Quantum espresso: a modular and open-source software project for quantum simulations of materials. J. Phys. Condens. Matter.

[39] Zhang J, Xie W, Zhao J, Zhang S (2017). Band alignment of two-dimensional lateral heterostructures. 2D Mater..

[40] Mott NF (1939). The theory of crystal rectifiers. Proceedings of the Royal Society of London A: Mathematical, Physical and Engineering Sciences.

[41] Yang S (2014). Direct observation of the work function evolution of graphene-two-dimensional metal contacts. J. Mater. Chem. C.

[42] Sup Choi, M. *et al*. Controlled charge trapping by molybdenum disulphide and graphene in ultrathin heterostructured memory devices. *Nat*. *Commun*. **4**, 1624 (2013). 10.1038/ncomms2652 23535645

[43] Zou X (2014). Interface engineering for high-performance top-gated mos_2_ field-effect transistors. Adv. Mater..

[44] Wu YQ (2008). Top-gated graphene field-effect-transistors formed by decomposition of sic. Appl. Phys. Lett..

[45] Liu W, Sarkar D, Kang J, Cao W, Banerjee K (2015). Impact of contact on the operation and performance of back-gated monolayer mos_2_ field-effect-transistors. ACS Nano.

[46] Chen, G. *Nanoscale Energy Transport and Conversion*, 1st edn (Oxford, 2005).

[47] Gilat G, Raubenheimer LJ (1966). Accurate numerical method for calculating frequency-distribution functions in solids. Phys. Rev..

[48] Datta, S. *Electronic Transport in Mesoscopic Systems*, 1st edn (Cambridge University Press, 1995).

[49] Datta, S. *Lessons from Nanoelectronics A New Perspective on Transport*, 1 (World Scientific Publishing Co. Pte. Ltd., 2012).

[50] Kochat V (2016). Magnitude and origin of electrical noise at individual grain boundaries in graphene. Nano Lett..

[51] Eberhart ME, Vvedensky DD (1987). Localized Grain-Boundary Electronic States and Intergranular Fracture. Phys. Rev. Lett..

[52] Luican-Mayer A (2016). Localized electronic states at grain boundaries on the surface of graphene and graphite. 2D Materials.

[53] Dutta S, Wakabayashi K (2015). Magnetization due to localized states on graphene grain boundary. Sci. Rep..

[54] Perdew JP, Zunger A (1981). Self-interaction correction to density-functional approximations for many-electron systems. Phys. Rev. B.

[55] Perdew JP, Wang Y (1992). Accurate and simple analytic representation of the electron-gas correlation energy. Phys. Rev. B.

[56] Sharvin YV, Bogatina NI (1969). Investigation of focusing of electron beams in a metal using longitudinal magnetic field. Sov. Phys. JETP.

[57] van Wees BJ (1988). Quantized conductance of point contacts in a two-dimensional electron gas. Phys. Rev. Lett..

[58] Wharam DA (1988). One-dimensional transport and the quantisation of the ballistic resistance. J. Phys. C.

[59] Lundstrom, M. & Jeong, C. *Near*-*equilibrium transport Fundamentals and applications*, 2 edn (World Scientific Publishing Co. Pte. Ltd., 2013).

[60] Yasaei, P. *et al*. Chemical sensing with switchable transport channels in graphene grain boundaries. *Nat*. *Commun*. **5**, 4911– (2014). 10.1038/ncomms5911 25241799

[61] Vlassiouk I (2011). Electrical and thermal conductivity of low temperature cvd graphene: the effect of disorder. Nanotechnology.

[62] Yagi K (2013). Dependence of field-effect mobility of graphene grown by thermal chemical vapor deposition on its grain size. Japanese Journal of Applied Physics.

[63] Duong DL (2012). Probing graphene grain boundaries with optical microscopy. Nature.

[64] Yang M, Sasaki S, Ohnishi M, Suzuki K, Miura H (2016). Electronic properties and strain sensitivity of cvd-grown graphene with acetylene. Japanese Journal of Applied Physics.

[65] Lee D (2014). Significant enhancement of the electrical transport properties of graphene films by controlling the surface roughness of cu foils before and during chemical vapor deposition. Nanoscale.

